# Dose-dependent mesothelioma induction by intraperitoneal administration of multi-wall carbon nanotubes in p53 heterozygous mice

**DOI:** 10.1111/j.1349-7006.2012.02318.x

**Published:** 2012-04-27

**Authors:** Atsuya Takagi, Akihiko Hirose, Mitsuru Futakuchi, Hiroyuki Tsuda, Jun Kanno

**Affiliations:** 1 Division of Cellular and Molecular Toxicology, Biological Safety Research Center, National Institute of Health Sciences, Tokyo; Japan; 2 Division of Risk Assessment, Biological Safety Research Center, National Institute of Health Sciences, Tokyo; Japan; 3 Department of Molecular Toxicology, Nagoya City University Graduate School of Medical Sciences; Nagoya, Japan; 4 Nanomaterial Toxicology Project Laboratory, Nagoya City University, Nagoya, Japan

## Abstract

Among various types of multi-wall carbon nanotubes (mwcnt) are those containing fibrous particles longer than 5 μm with an aspect ratio of more than three (i.e. dimensions similar to mesotheliomagenic asbestos). A previous study showed that micrometer-sized mwcnt (μm-mwcnt) administered intraperitoneally at a dose of 3000 μg/mouse corresponding to 1 × 10^9^ fibers per mouse induced mesotheliomas in p53 heterozygous mice. Here, we report a dose-response study; three groups of p53 heterozygous mice (*n* = 20) were given a single intraperitoneal injection of 300 μg/mouse of μm-mwcnt (corresponding to 1 × 10^8^ fibers), 30 μg/mouse (1 × 10^7^) or 3 μg/mouse (1 × 10^6^), respectively, and observed for up to 1 year. The cumulative incidence of mesotheliomas was 19/20, 17/20 and 5/20, respectively. The severity of peritoneal adhesion and granuloma formation were dose-dependent and minimal in the lowest dose group. However, the time of tumor onset was apparently independent of the dose. All mice in the lowest dose group that survived until the terminal kill had microscopic atypical mesothelial hyperplasia considered as a precursor lesion of mesothelioma. Right beneath was a mononuclear cell accumulation consisting of cd45- or cd3-positive lymphocytes and cd45/cd3-negative f4/80 faintly positive macrophages; some of the macrophages contained singular mwcnt in their cytoplasm. The lesions were devoid of epithelioid cell granuloma and fibrosis. These findings were in favor of the widely proposed mode of action of fiber carcinogenesis, that is, frustrated phagocytosis where the mesotheliomagenic microenvironment on the peritoneal surface is neither qualitatively altered by the density of the fibers per area nor by the formation of granulomas against agglomerates. (*Cancer Sci* 2012; 103: 1440–1444)

Unique properties such as persistency and electric conductivity promise a high potential for technology applications of carbon nanotubes (e.g. in lithium ion batteries). Immediately after the invention of the carbon nanotube, its persistency and fibrous shape have posed a challenge for toxicology known as “fiber carcinogenesis”.^1^ A recent study showed that a particular type of multi-wall carbon nanotube (Mitsui MWCNT-7, designated in general here as micrometer-sized MWCNT or μm-MWCNT) contains a considerable percentage of particles similar to asbestos in length and diameter.^2^ To investigate its mesotheliomagenic potential, we used an intraperitoneal injection (i.p.) method that was extensively used in the 1970s and 1980s for the elucidation of key dimensions of the fiber (e.g. length and diameter) and for toxicity assessment of various man-made fibers.^3^^–^6 Although the route of exposure is not realistic for humans, the i.p. injection method has been considered appropriate to assess the mesotheliomagenic potential of fibers,^7^ and the least potent fibers were found to induce a positive result at a dose of 10^9^ fibers i.p. in rats.^6^^–^8

Our first study identified the mesotheliomagenic potency of Mitsui MWCNT-7 at a single maximum dose (i.e. 10^9^ fibers) in the peritoneal cavity of p53 heterozygous (p53+/−) mice^2^ (data shown as a reference in Fig 1). Marsella *et al*.^9^ has shown that development of mesothelioma by crocidolite asbestos was accelerated in this mutant mouse. We have bred this mouse and tested it as an alternative model to replace the wild-type mouse carcinogenicity test of the National Toxicology Program of the National Institute of Environmental Health Sciences/NIH of the United States.^10^ As a result, spontaneous neoplastic lesions of this model have been well characterized.^11^

**Figure 1:**
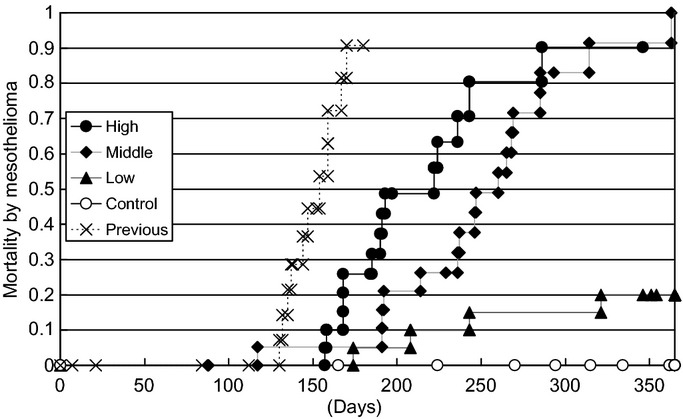
Dose-dependent induction of mesotheliomas by micrometer-sized multi-wall carbon nanotubes (μm-mwcnt). Mice with lethal mesotheliomas are plotted using the Kaplan–Meier method. High: 300 μg/mouse, corresponding to 1 × 10^8^ fibers/mouse; middle: 30 μg/mouse, corresponding to 1 × 10^7^ fibers/mouse; low: 3 μg/mouse, corresponding to 1 × 10^6^ fibers/mouse); previous: data from a previous study (i.e. 3 mg/mouse, corresponding to 1 × 10^9^ fibers/mouse). No mesothelioma was observed in the vehicle control group.

Here, we applied the same fiber to p53+/− mice at doses of 1/10, 1/100, and 1/1000 of the dose used in the previous study (i.e. 300, 30 and 3 μg/mouse), which corresponds to approximately 1 × 10^8^, 1 × 10^7^, and 1 × 10^6^ fibers per mouse, respectively, and monitored the mice for 1 year.

## Materials and Methods

### Experimental animals

The p53+/− mice were generously supplied by Dr S. Aizawa,^12^ and back crossed with normal wild-type C57BL/6 females (SLC, Shizuoka, Japan) for more than 20 generations at the National Institute of Health Sciences (NIHS), Tokyo. Eighty male p53+/− mice aged 9–11 weeks were divided into four groups of 20 mice, and housed individually under specific pathogen-free conditions with a 12-h light-dark cycle at a NIHS animal facility. They were given tap water and autoclaved CRF-1 pellets (Oriental Yeast Co. Ltd., Tokyo, Japan) *ad libitum*. Experiments were humanely conducted under the regulation and permission of the Animal Care and Use Committee of the NIHS.

### Histology

Liver, kidney, spleen, lung, digestive tract and macroscopic tumors (*en bloc* in the case of severe peritoneal adhesion) were fixed in 10% neutral buffered formalin. After conventional processing, paraffin-embedded sections were stained with hematoxylin–eosin (HE) and examined histopathologically under a light microscope. A pair of polarizing filters was set to a light microscope to detect birefringent particles.

For the selected atypical mesothelial hyperplasia lesions, serial sections were stained for CD45R(B220), CD3 and F4/80 using anti-mouse CD45R (eBioscience, San Diego, CA, USA), anti-rat CD3 (AbD Serotec, Kidlington, UK), anti-mouse F4/80 antibodies (eBioscience), which were diluted at 1:100, 1:50 and 1:50, respectively. The slides were incubated at 4°C overnight and then incubated for 1 h with biotinylated species-specific secondary antibodies diluted 1:500 (Vector Laboratories, Burlingame, CA, USA) and visualized using avidin-conjugated alkaline phosphatase complex (ABC kit; Vector Laboratories).

### Test material

Multi-wall carbon nanotube (MITSUI MWCNT-7, Lot No. 060125-01k), the same lot used in our previous study^2^ was used. As reported in our previous paper, one gram of MWCNT corresponded to 3.55 × 10^11^ particles. The length ranged from 1 to 20 μm with a median of 2 μm. More than 25% of the particles were longer than 5 μm; their width ranged from 70 to 170 nm with a median of 90 nm. The approximate average content of iron was 3500 ppm (0.35%) and that of sulfur was 470 ppm. The concentration of chlorine in the fibers was 20 ppm and that of fluorine and bromine was below the limits of detection (5 and 40 ppm, respectively).^2^

Multi-wall carbon nanotubes was suspended at a concentration of 3 mg/mL to 0.5% methyl cellulose (Shin-Etsu Chemical Co. Ltd, Tokyo, Japan) solution and autoclaved (121°C, 15 min). After addition of Tween 80 (Tokyo Chemical Industry Co. Ltd, Tokyo, Japan; final 1.0% concentration), the solution was subjected to sonication at 150 watt for 5 min using an ultrasonic homogenizer (VP30s; TAITEC Co., Saitama, Japan).

### Treatment

Eighty male p53+/− mice aged 9–11 weeks were randomly divided into four groups of 20. The high-dose group mice were given a single i.p. injection of 300 μg/mouse of MWCNT particles (corresponding to 1 × 10^8^ fibers) in 1 mL suspension. The middle-dose group mice received 30 μg/mouse (1 × 10^7^) and the low-dose group mice received 3 μg/mouse (1 × 10^6^), respectively. The control group mice received vehicle solution (1 mL). Treated mice were monitored for 1 year. To minimize stress to the animals and re-aggregation of suspension, the injection was promptly performed without anesthesia.

## Results

**Figure 2:**
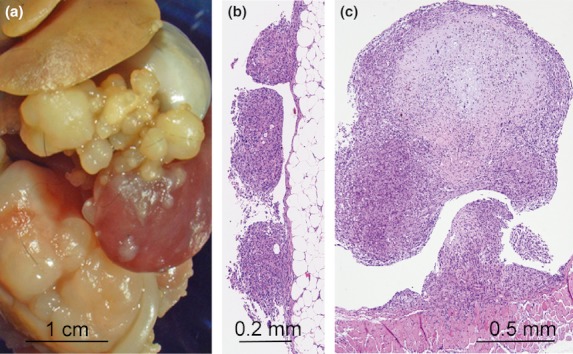
Morphology of the induced mesotheliomas in the low-dose group. (a) Macroscopic view of the abdominal cavity of a mouse in the low-dose group. Multiple nodules are seen on the surface of the peritoneal serosa. This mouse died on day 243 with multiple nodules up to size 1 × 1 × 1 cm. (b) Low-power light microscopy view of the multiple nodules on the peritoneal surface of the mesentery. Granulomas and fibrous scars are minimal in the low-dose group. (c) Histology of a small nodule compatible with a diagnosis of moderately to poorly differentiated epithelioid mesothelioma. Larger nodules tended to be composed of undifferentiated sarcomatous components.^2^

**Figure 3:**
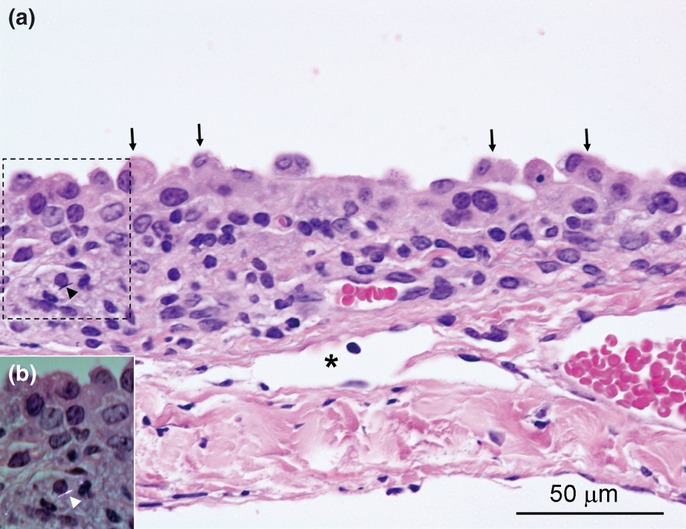
Atypical mesothelial hyperplasia. (a) Atypical mesothelial hyperplasia of the tendinous portion of the diaphragm of a mouse in the low-dose group (sampled at terminal kill, that is, 365 days after i.p. inoculation of the multi-wall carbon nanotubes [mwcnt]). Arrows: hobnail appearance of the atypical hyperplastic mesothelial cells; asterisk: lymphatic drainage of the peritoneal cavity. (b) Polarized image of the dotted area in (a). Arrowhead: a mwcnt fiber in a macrophage-like cell (birefringent).

**Figure 4:**
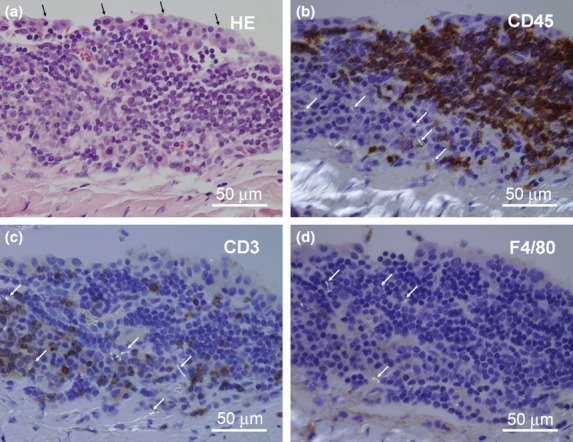
Immunohistochemistry of lentiform mononuclear cell accumulation underlying the atypical mesothelial hyperplasia. (a) Serial section of an atypical mesothelial hyperplasia of the tendinous portion of the diaphragm of a mouse in the low-dose group (sampled at terminal kill). (a) Hematoxylin–eosin staining. Black arrows: hobnail appearance of the hyperplastic mesothelial cells. (b–d) Polarized image of the serial sections immunohistochemically stained for cd45, cd3 and F4/80. Multi-wall carbon nanotubes (birefringent; white arrows) are seen in the macrophage-like cd45/cd3-negative, F4/80-faintly positive cell cytoplasm. It is noted that epithelioid cell granuloma and fibrous scars are absent in this type of lesion.

Peritoneal mesotheliomas were induced in a dose-dependent manner shown by an increase in the cumulative incidence of the tumors (Fig. 1). In the high-dose group, 14/20 mice had single or multiple lethal mesotheliomas up to 2 × 2 cm in size located within the peritoneal cavity, invading adjacent organs and structures with or without peritoneal dissemination. The remaining mice died of ileus due to severe peritoneal adhesion and fibrosis, and among them five had small incidental (non-lethal) mesotheliomas. The total incidence of mesothelioma was 19/20 (95%) among the animals. These lesions were qualitatively identical to our previous study.^2^ In the middle-dose group, 17/20 (85%) mice had lethal mesothelioma. Three mice without lethal mesothelioma died or became moribund due to other reasons including leukemia. In the low-dose group, 4/20 mice had lethal mesothelioma (Fig. 2) and 1/20 had a non-lethal mesothelioma (found at the terminal kill on day 365), which makes the overall incidence of mesothelioma 5/20 (25%). The other 15 mice that survived until the terminal kill showed focal mesothelial atypical hyperplasia.^13^ These lesions, up to 0.5 mm in diameter, consisted of a single layer of mesothelium characterized by cuboidal or hobnail appearance with slight to moderate nuclear atypia. Right beneath the atypical mesothelium was a lentiform accumulation of mononuclear inflammatory cells up to 0.1 mm in thickness (Fig. 3). The accumulation is a combination of ill-demarcated zones of CD45-positive lymphocytes, CD3-positive lymphocytes and CD45/CD3-negative F4/80-negative or CD45/CD3-negative F4/80 weakly positive macrophage-like cells (Fig. 4). Single MWCNT fiber was often found in the cytoplasm of the macrophage-like cells. These lesions were devoid of epithelioid cell granuloma and fibrous scars.

Peritoneal fibrosis, peritoneal adhesion and formation of foreign body granulomas towards agglomerated MWCNT were dose dependent and minimal in the low-dose group. In the control group, mesotheliomas were not found (0%). There were eight mice with lethal or incidental thymic lymphoma, leukemia or reticulum cell sarcoma, osteosarcoma of the cranial bone, and 12/20 were tumor free. These tumors are known to develop spontaneously in p53+/− mice with increasing age^10^ and none of these tumors were treatment dependent.

Histology of the mesotheliomas ranged from a differentiated epithelioid type to an undifferentiated sarcomatous type. Osteoid and rhabdoid differentiations, both known in human cases,^14^^–^16 were found in nine mice (two in the low dose, three in the middle dose, and four in the high dose group, respectively) among a total of 41 mesothelioma cases in the present study.

**Figure 5:**
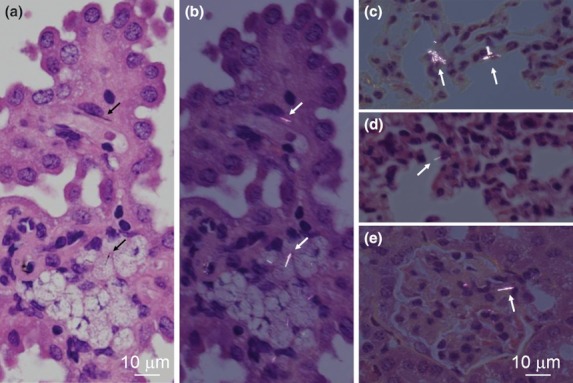
Systemic distribution of singular fibers. Single micrometer-sized multi-wall carbon nanotube fibers are found in the choroid plexus in (a) normal lighting and (b) polarized light (in a mouse from the high-dose group sampled on day 168), (c) lung as an agglomerate within macrophages (polarized light) or (d) as singular fibers (polarized light) and (e) a renal glomerulus (polarized light) (in a mouse from the high-dose group sampled on day 197). Fibers were also found in hepatic sinusoids and mesenteric lymph nodes (not shown).

An additional finding was the dissemination of singular fibers to systemic organs such as the liver, mesenteric lymph nodes, pulmohilar lymph nodes, choroid plexus of the brain, glomeruli of the kidney and lung alveoli (Fig. 5). Because the brain, including the choroid plexus, lacks afferent lymphatics,^17^,^18^ it is probable that the fibers were distributed systemically via the blood stream.

## Discussion

The present study showed a dose-dependent induction of mesothelioma by the μm-MWCNT from 1/1000 of the dose of our previous study (i.e. 3 μg/mouse corresponding to 1 × 10^6^ fibers).

It is noted that the mesotheliomas of the low-dose group were not accompanied by foreign body granulomas or fibrous scars. The mesothelial atypical hyperplasia found 1 year after the i.p. injection in the low-dose group mice were also devoid of foreign body granulomas and fibrous scars. Instead, these lesions were backed up by an accumulation of mononuclear inflammatory cells. The macrophage-like cells in the accumulation, negative to weakly positive for F8/40, were often positive for singular MWCNT in their cytoplasm. As the mesothelial atypical hyperplasia is considered as precancerous lesions, the essential background of mesotheliomagenesis might be the inflammatory lesions without granulomas and fibrous scars formed against MWCNT agglomerates. The mesothelial atypical hyperplasia can be regarded as a lesion driven by the frustrated phagocytosis against MWCNT.

In general, carcinogenesis is considered a multistage process. In the case of chemical carcinogens with clear genotoxic properties, tumor onset occurs significantly earlier at higher doses.^19^,^20^ Presumably, an increasing number of hits to a target cell leads to faster progression of the carcinogenic stages. Here, in contrast, the onset time of the mesotheliomas was apparently dose independent. Onset estimates calculated as x-intercepts of logarithmic approximation^21^,^22^ were 126, 146, 148 and 138 days for the previous study data^2^ and the three doses of the present study, respectively (Fig. S1). Mechanistically, a direct effect to a mesothelial cell, such as mutagenic or clastogenic effect, would favor a dose-dependent acceleration of the onset. If the granulomas are an important promoting factor of mesotheliomagenesis,^23^ the highest dose group should have had the earliest onset because the granuloma formation can take place within 7 days subsequent to the i.p. injection.^24^ In contrast, the humoral stimuli released from the nearby macrophages in the condition of frustrated phagocytosis^25^ would match with this finding. As shown in Figures 3 and 4, the reactive mesothelial cells are accompanied by mononuclear inflammatory cells with MWCNT fibers, but not by epithelioid cell granulomas or fibrous scars. One could speculate that each loci of frustrated phagocytosis could continuously stimulate the nearby mesothelial cells, that is, first to induce reactive hyperplasia and then as the next step proceed towards mesothelioma. If the dose is the determinant of the number of such loci within a defined surface area of peritoneal mesothelial membrane, then it is natural to predict that the earliest day of tumor onset is dose independent, whereas the probability of tumor onset closer to the earliest day will increase in a dose-dependent manner.

An additional finding was the distribution of singular fibers to systemic organs such as the liver, mesenteric lymph nodes, pulmohilar lymph nodes, choroid plexus of the brain, glomeruli of the kidney and lung alveoli (Fig. 5). Because the brain, including the choroid plexus, lacks afferent lymphatics,^17^,^18^ it is probable that the fibers were distributed systemically via the blood stream. Its importance to human health could be closely linked to the systemic distribution of asbestos reported in humans,^26^,^27^ that is, a possibility of increasing systemic diseases such as cancer in various organs^28^ and autoimmune diseases.^29^*In vivo* studies on the shorter fractions of MWCNT for its systemic toxicity would be essential.

It is likely that the peritoneal cavity served as a filter to segregate large agglomerates from the i.p. injected MWCNT suspension by the formation of foreign body granulomas and fibrous scars, leaving singular long MWCNT fibers for mesotheliomagenesis (frustrated phagocytosis) and short singular fibers for systemic distribution. The short fibers might have passed through the stomata (pores) of the mesothelium^23^ or been transported by macrophages into lymphatics and to the vascular systems. As a whole, the i.p. injection model appears to be a robust system for the hazard identification of fiber carcinogenesis of asbestos-like fibrous particulate matter and of systemic toxicity of fibrous and non-fibrous particulate matter including nanoparticles that can enter the blood stream.

In conclusion, μm-MWCNT was mesotheliomagenic in the p53+/− mouse peritoneal cavity model in a dose-dependent manner from as low as 3 μg per mouse or approximately 10^6^ fibers per mouse. Although the molecular mechanisms of fiber mesotheliomagenesis are unknown, the minute lesions seen in the lowest dose group and the dose-response characteristics might be consistent with the concept of frustrated phagocytosis and also with the observation in human asbestos epidemiology that there would be no practical threshold for fiber mesotheliomagenesis.

## Supporting Information

Fig. S1 Estimation of the time of tumor onset.
