# Supplementary material for: Dose-dependent mesothelioma induction by intraperitoneal administration of multi-wall carbon nanotubes in p53 heterozygous mice
Source: Cancer Sci. 2012 Apr 27;103(8):1440–4. doi: 10.1111/j.1349-7006.2012.02318.x (PMC3569866; doi:10.1111/j.1349-7006.2012.02318.x)
Supplement: Fig. S1 — Estimation of the time of tumor onset. [file cas0103-1440-SD1.pdf]

Supplement material

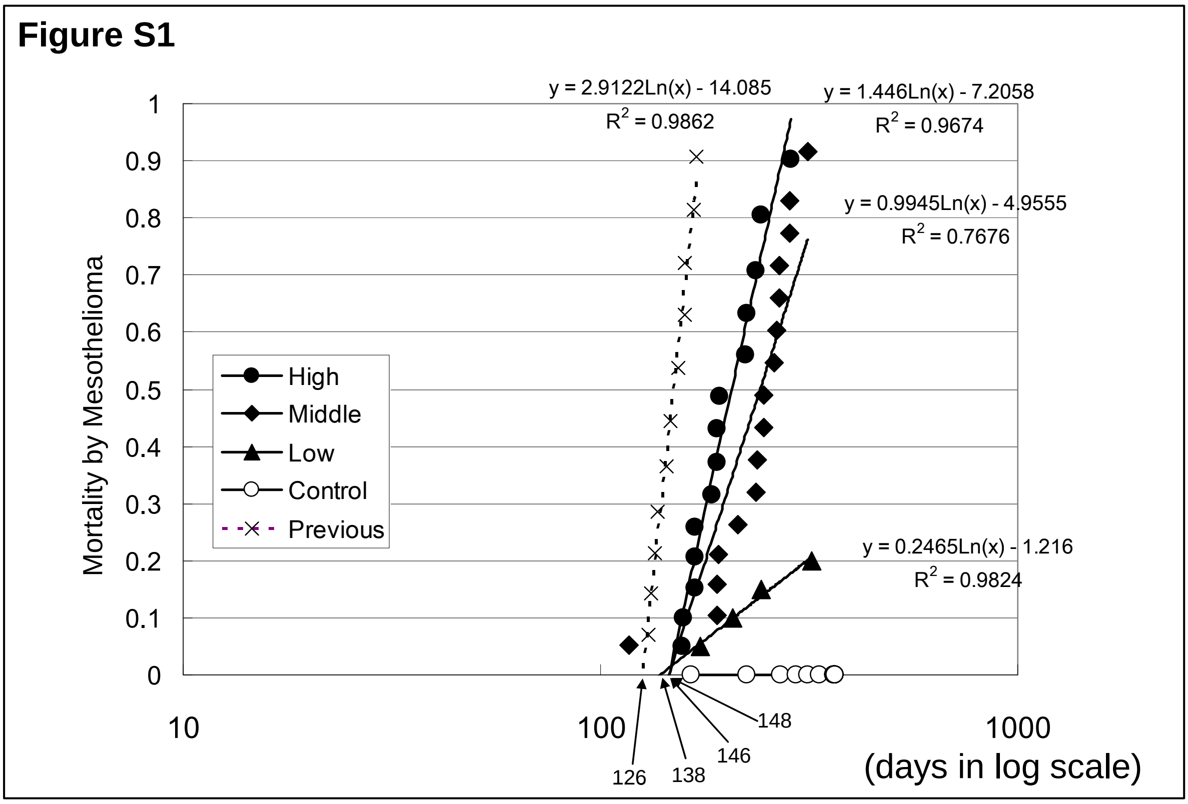

Figure S1. Estimation of the time of tumor onset.

The time of onset of the mesothelioma of each group was estimated by the x-intercepts of logarithmic approximation. The estimated onset was 126, 146, 148 and 138 days for the previous study data and the three doses of this study, respectively. The fitted functions and r^2 values are shown in the figure.
